# Retinal compensatory changes after light damage in albino mice

**Published:** 2012-03-24

**Authors:** Luis Montalbán-Soler, Luis Alarcón-Martínez, Manuel Jiménez-López, Manuel Salinas-Navarro, Caridad Galindo-Romero, Fabrízio Bezerra de Sá, Diego García-Ayuso, Marcelino Avilés-Trigueros, Manuel Vidal-Sanz, Marta Agudo-Barriuso, Maria P. Villegas-Pérez

**Affiliations:** 1 Departamento de Oftalmología, Optometría, Otorrinolaringología y Anatomía Patológica, Facultad de Medicina, Universidad de Murcia, Murcia, Spain; 2 Unidad de Investigación. Hospital Universitario Virgen de la Arrixaca. Fundación para la Formación e Investigación Sanitarias de la Región de Murcia, Murcia, Spain; 3 Universidade Federal Rural de Pernambuco UFRPE. Rua Dom Manoel de Medeiros, s/n, Dois Irmãos, Brasil

## Abstract

**Purpose:**

To investigate the anatomic and functional changes triggered by light exposure in the albino mouse retina and compare them with those observed in the albino rat.

**Methods:**

BALB/c albino mice were exposed to 3,000 lx of white light during 24 h and their retinas analyzed from 1 to 180 days after light exposure (ALE). Left pupil mydriasis was induced with topical atropine. Retinal function was analyzed by electroretinographic (ERG) recording. To assess retinal degeneration, hematoxylin and eosin staining, the TdT-mediated dUTP nick-end labeling (TUNEL) technique, and quantitative immunohistofluorescence for synaptophysin and protein kinase Cα (PKCα) were used in cross sections. Intravenous injection of horseradish peroxidase and Fluoro-Gold™ tracing were used in whole-mounted retinas to study the retinal vasculature and the retinal ganglion cell (RGC) population, respectively.

**Results:**

Light exposure caused apoptotic photoreceptor death in the central retina. This death was more severe in the dorsal than in the ventral retina, sparing the periphery. Neither retinal vascular leakage nor retinal ganglion cell death was observed ALE. The electroretinographic a-wave was permanently impaired, while the b-wave decreased but recovered gradually by 180 days ALE. The scotopic threshold responses, associated with the inner retinal function, diminished at first but recovered completely by 14 days ALE. This functional recovery was concomitant with the upregulation of protein kinase Cα and synaptophysin. Similar results were obtained in both eyes, irrespective of mydriasis.

**Conclusions:**

In albino mice, light exposure induces substantial retinal damage, but the surviving photoreceptors, together with compensatory morphological/molecular changes, allow an important restoration of the retinal function.

## Introduction

The rodent retina is widely used to investigate retinal diseases, as well as the response of central nervous system neurons to injury. Light-induced retinal damage (phototoxicity) selectively brings about photoreceptor cell death. Thus, this model is useful for studying the potential mechanisms underlying photoreceptor death and the subsequent retinal degeneration processes [1,2], since it mimics the photoreceptor degeneration that forms the main characteristic of human diseases such as retinitis pigmentosa or age-related macular degeneration [3,4].

In albino and pigmented rats, our group has shown that phototoxicity initially causes vascular leakage in an “arciform area” located in the mid-dorsal retina. Photoreceptor death that is at least in part apoptotic commences in this area first, before spreading with time to the rest of the retina. Secondary to photoreceptor degeneration, the inner retina undergoes degenerative changes that end in the death of retinal ganglion cells (RGCs) [5,6]. In addition, the electroretinographic (ERG) response is abolished permanently in albino rats [5].

The ERG test analyzes the functionality of the different neuronal populations of the retina. The a-wave is due mainly to photoreceptor activity, while the b-wave is due to the activity of second- and third-order retinal neurons [7,8]. Recent studies have documented that in different animals, including rodents, the scotopic threshold response (STR, a third ERG wave with a positive and negative component) reflects the activity of the innermost retinal cells, mainly RGCs and amacrine cells [9-15]. In rodent models of light-induced damage to the retina, ERG is used mainly to study the functionality of photoreceptors or bipolar cells [16]. However, there is a report describing an impairment of the STR wave in the rat retina after phototoxic insult [17] that provides electrophysiological corroboration of our previous work documenting morphological alterations and neuronal death in the inner rat retina after phototoxicity [5,6].

Our group has also shown that axonal alterations and RGC death occur in mice and rats suffering inherited photoreceptor degeneration [6,18-22]. These animals have been widely used as models for human photoreceptor diseases [2,23-25]. Because we noticed in our earlier work that there were obvious differences in RGC degeneration between rats and mice with inherited retinal degeneration, and because we already know the degenerative events taking place in the RGC layer of the albino/pigmented rat retina after light exposure (ALE) [5,6], we have now investigated the effects of phototoxicity on the inner retina of the albino BALB/c mouse to compare it with the rat model.

In particular, in this study we have analyzed: i) the temporal and spatial loss of photoreceptors; ii) whether their death is apoptotic; iii) the effect of phototoxicity on vascular leakage; iv) whether phototoxicity induces loss of RGCs; v) the function of the inner and outer retina (by analyzing the STR and a- and b-waves, respectively); and finally; vi) the expression pattern and levels of two proteins associated with the mechanisms that generate ERG responses. This latter experiment allowed us to assess the possible compensatory changes that have taken place in the retina after phototoxicity.

## Methods

### Animal handling

Two-month-old female BALB/cAnNHsd albino mice from Harlan (Barcelona, Spain) were used. All animals were treated according to our institutional guidelines, the European Union regulations, and the Association for Research in Vision and Ophthalmology Guidelines for the use of animals in research.

Before light exposure, the animals were reared in cages containing four animals, fed ad libitum, and kept in a 12h:12h light-dark cycle under controlled temperature (22–24 °C) and humidity in the viverium of the University of Murcia. Light intensity within the cages oscillated between 5 and 50 lx.

Animals were studied at 1 (n=8), 3 (n=8), 7 (n=16), 14 (n=12), 30 (n=13), 90 (n=10), or 180 (n=10) days ALE. The ERG responses obtained from groups processed 30 days or earlier ALE were compared to their baseline responses recorded before light exposure. The ERG responses from groups processed 90 or 180 days ALE were compared to those obtained from age-matched control animals to take into account the effect of age on retinal function.

For all manipulations and experimental procedures (except light exposure), the animals were anesthetized with an intraperitoneal injection of a mixture of ketamine (70 mg/kg Ketolar®, Pfizer, Alcobendas, Madrid, Spain) and xylazine (10 mg/kg Rompun®, Bayer, Kiel, Germany) in 0.1 ml of saline.

### Light exposure

Before light exposure, left pupil mydriasis was induced by topical application of a drop of 1% atropine (Colirio de atropina 1%®; Alcon S.A., Barcelona, Spain). The right eye was not dilated to be used for comparison.

Because the severity of retinal phototoxicity in rodents depends on the time of the day when exposure starts (circadian rhythm) and on the previous light exposure (rearing conditions and dark adaptation period) [3,26-28], the exposure always began between 10 and 12 AM and after 12 h of dark adaptation.

Animals were individually exposed to 24 h of continuous fluorescent cold white light. Fluorescent bulbs were situated in the ceiling right above the animal cages, which were transparent. Light intensity within the cages was 3,000±100 lx. Mydriasis was inspected 12 h after the initiation of light exposure, and when necessary, a second drop of atropine was instilled in the left eye at this time.

### Electroretinography

ERG recordings were performed as previously described [10]. Briefly, animals were dark adapted overnight before ERG recordings, and their manipulation was performed under dim red light (λ>600 nm). Mice were anesthetized and bilateral pupil mydriasis was induced by topical application of a drop of 1% tropicamide (Colirio de tropicamida 1%®; Alcon, S.A., El Masnou, Barcelona, Spain) in both eyes; the animals’ body temperature was kept at 37 °C by placing them on top of a water heat pad (TP500 T/Pump; Gaymar Industries, Orchard Park, NY). For light stimulation, a Ganzfeld dome and multiple reflections of light generated by light-emitting diodes were used. For high-intensity illuminations, a single light-emitting diode placed close (1 mm) to the eye was used. Light intensity was calibrated by a dual-biosignal generator device specifically adapted for ERG responses. ERG recordings were made using Burian-Allen bipolar electrodes shaped as a corneal contact lens (Hansen Labs, Coralville, IA). The electrical signals generated in the retina were amplified (×1,000) and filtered (band pass from 1 Hz to 1,000 Hz) with a commercial amplifier (Digitimer Ltd., Letchworth Garden City, UK). The recorded signals were digitized (Power Lab; ADInstruments Pty. Ltd., Chalgrove, UK) and displayed on a personal computer. ERG recordings were taken simultaneously from both eyes. Light stimuli were calibrated before each experiment to assure identical recording parameters for both eyes. The retina was stimulated using light intensities ranging between 10^−5^ and 10^−4^ cd·s·m^−2^ for the STR, 10^−4^ and 10^−2^ cd·s·m^−2^ for the response mediated by rods, and between 10^−2^ and 10^2^ cd·s·m^−2^ for the mixed response (response mediated by rods and cones). The animals were then light adapted for 5 min and a maximum intensity stimulus (10^2^ cd·s·m^−2^) was used to obtain the photopic response (mediated by cones). For each light stimulus, a series of ERG responses was averaged and the interval between light flashes was adjusted to allow response recovery between stimuli. After each session, tobramycin ointment (Tobrex®; Alcon, S.A., El Masnou, Barcelona, Spain) was applied on both corneas to prevent corneal desiccation during recovery from anesthesia. Recording analysis was performed using the normalization criteria established by the International Society for Clinical Electrophysiology of Vision.

### Horseradish peroxidase injection: vascular leakage

Four control animals and various experimental animals that were processed 1 (n=4), 3 (n=4), 7 (n=4), 14 (n=4), and 30 (n=4) days ALE received, 10 min before processing, an injection in the inferior cava vein of 0.1 ml of horseradish peroxidase (100 μg/μl diluted in saline; HRP; type I, MW 44 kDa, Sigma P 8125, Steimheim, Germany).

### Fluoro-Gold application: retinal ganglion cell tracing

In four control animals and various experimental animals that were processed 3 (n=8) and 6 months (n=4) ALE, Fluoro-Gold (FG) was applied to both superior colliculi 7 days before processing, following the techniques already described [29,30]. Briefly, animals were anesthetized, the mid-brain was exposed and, after removing the pia mater overlying the superior colliculi, a piece of sponge (Spongostan Film, Ferronsan, Denmark) soaked in 3% FG diluted in 10% dimethyl sulfoxide-saline (Sigma Aldrich, Madrid, Spain) was applied over the surface of both superior colliculi. FG is incorporated to the axon terminals and retrogradely transported to the retina where it accumulates in the RGC somas.

### Tissue processing

Animals were sacrificed with an intraperitoneal lethal dose (0.4–0.5 ml) of 20% sodium pentobarbital (Dolethal®; Vetoquinol S.A., Lure, France). The eyes were enucleated and processed to obtain either retinal whole mounts or cross sections.

### Whole mounts

Horseradish peroxidase–injected animals. Eyes were enucleated and immersed for 1 h in 4% paraformaldehyde in 0.1M PBS (pH 7.2–7.4). Later, the retinas were dissected as whole mounts by making four radial cuts in the superior, inferior, temporal, and nasal retina. To maintain their orientation, the deepest cut was made in the superior retina. The retinas were then postfixed for 1 h in the same fixative solution, rinsed in PBS, reacted for HRP demonstration using a modified Hanker-Yates reaction [31] to visualize the retinal vasculature [5,6,18,20,32], rinsed, and mounted on gelatin-coated slides, vitreal side up, with a small quantity of a solution of 50% glycerol in PBS.

Fluoro-Gold-traced animals. Animals were first perfused through the left ventricle with saline and subsequently with 4% paraformaldehyde at 4 °C. The eyes were enucleated and processed in the same manner as the eyes of the HRP-injected animals. The whole mounts were immediately mounted as before, and observed and photographed with a fluorescence microscope (Axioscop 2 Plus; Zeiss, Jena, Germany).

### Cross sections

Retinal cross sections were obtained from control (n=5) and experimental animals processed 1 (n=5), 3 (n=8), 7 (n=5), 14 (n=4), 30 (n=7), 90 (n=6), or 180 (n=5) days ALE. After perfusion, as above, the eyes were enucleated and the superior portion of the sclera and the superior rectus muscle marked with china ink to maintain their orientation. The cornea and lens were removed and the eyecups were postfixed for 48 h in the same fixative, dehydrated through alcohols and 1-butanol, and embedded in paraffin. Three-micron-thick cross sections were obtained in a rotational microtome (Microm HM-340-E; Microm Laborgerate GmbH, Walldorf, Germany) and mounted on slides coated with 0.01% poly-L-lysine (Sigma-Aldrich, St. Louis, MO). Only the sections containing the optic nerve head were kept and stained with hematoxylin and eosin, or processed for TdT-mediated dUTP nick-end labeling (TUNEL) or immunohistofluorescence.

### Hematoxylin and eosin staining

A series of sections (4–6 per animal) were deparaffinized, rehydrated, stained with Hansen’s hematoxylin and eosin, and mounted with DePex (BDH Laboratory Supplies, Poole, UK).

### TdT-mediated dUTP nick-end labeling

Some sections (4–6 per animal) were used to detect apoptotic nuclei using the TUNEL method. TUNEL assay was performed according to the manufacturer’s protocol (FragEL™ DNA Fragmentation Detection Kit, Qiagen, Merck Bio, Nottingham, UK) with slight modifications as follows: Biotin-labeled DNA was detected by 2 h incubation at room temperature with avidin- tetra-methyl-rhodamine isothiocyanate (TRITC; Sigma-Aldrich, Madrid, Spain) diluted 1:500 in PBS containing 0.1% Triton. After washing, slides were mounted with antifading medium containing 4',6-diamidino-2-phenylindole (DAPI; VectaShield Mounting Medium with DAPI, Vector, Atom, Alicante, Spain) to counterstain all retinal nuclei.

### Immunohistofluorescence

Additional sections (4–6 per animal) were washed in PBS, immersed in citrate buffer (pH 6.0), and heated for 10 min in a microwave for antigen retrieval. They were then cooled at room temperature for 20 min, washed in PBS, and processed for immunohistofluorescence. In brief, the sections were incubated overnight at 4 °C with either mouse anti–protein kinase Cα (PKCα; 1:250; Santa Cruz Biotechnology, Heidelberg, Germany) or mouse anti-synaptophysin (1:250; Sigma-Aldrich, Madrid, Spain) diluted in blocking buffer (0.5% TritonX-100 and 10% normal goat serum in PBS). Secondary detection was performed using Alexa Fluor-488 goat anti-mouse or Alexa Fluor-568 goat anti-mouse (Molecular Probes, Invitrogen, Barcelona, Spain), each diluted 1:500 in PBS–0.5% Triton. Finally, all sections were thoroughly washed in PBS and mounted with a small amount of antifading medium (Vectashield® Mounting Medium; Vector; Atom, Alicante, Spain). Negative controls were obtained by the suppression of primary antibodies.

### Image analysis

Retinas were observed and photographed with a light/fluorescence microscope (Axioscop 2 Plus; Zeiss) equipped with different fluorescence filters, a digital high-resolution camera (ProgRes^TM^ c10; Jenoptic, Jena, Germany), and a motorized stage (ProScan^TM^ H128; Prior Scientific Instruments Ltd., Cambridge, UK) connected to an image analysis system (Image-Pro Plus 5.1 for Windows®; Media Cybernetics, Silver Spring, MD) with an automatic frame-grabber device (Scope-Pro 5.0 for Windows®; Media Cybernetics). Individual frames were taken from areas of interest in some cross sections. Photomontages of whole mounted retinas or ocular cross sections were also obtained. These images were used for qualitative or quantitative analysis, and in some cases (see below) were automatically analyzed.

Hematoxylin and eosin–stained cross sections were qualitatively analyzed. In addition, the thickness of the outer nuclear layer (ONL) was quantified in these sections by counting the number of nuclei rows at eight equidistant retinal locations (four dorsal and four ventral), taking the optic nerve as reference (location 0). This analysis was performed in the right and left retinas of three control and three experimental animals processed at different times ALE. Three retinal cross sections were quantified per retina.

Using two different fluorescence filters, TUNEL and DAPI signals were acquired from the same representative regions of the peripapillary retina. Gross numbers of TUNEL-labeled nuclei were qualitatively analyzed in these photographs. To obtain composites of these photographs, TUNEL images were coupled using the “dissolve” option of the Adobe® Photoshop® (Adobe Systems Inc., San Jose, CA) CS3 software. This option gives an artificial signal, and thus the TUNEL red fluorescence is enhanced.

### Automated quantification of Fluoro-Gold-traced retinal ganglion cells in whole mounts

Photomontages of FG-traced retinal whole mounts were generally composed of 140 individual rectangular images (0.627 mm^2^ each). In each frame, RGCs were automatically counted to obtain a total number of RGCs per retina using previously published methods [33]. Briefly, we used Image-Pro® Plus (version 5.1 for Windows™; Media Cybernetics Inc., Bethesda, MD) macro language to apply a sequence of filters and transformations to each image to clarify cell limits and to separate individual cells for automatic cell counting. Each frame was also divided into 64 rectangular areas in which the density of FG-traced RGCs was calculated, color-coded, and graphically represented to obtain an isodensity map for each retina, using methods that have been previously described [33]. Briefly, we developed a specific subroutine using Image-Pro® Plus macro language in which every retinal frame was divided into 64 equally-sized rectangular areas of interest (AOI). In each AOI, the RGC number was obtained and the cell density was calculated. RGC densities per AOI were exported to a spreadsheet (Microsoft Office Excel 2003; Microsoft Corporation, Redmond, WA) and finally, data were represented as filled contour plots using a graphic software (Sigmaplot® 9.0; Systat Software Inc., Richmond, CA).

### Automatic measurement of fluorescence in cross sections

Measurement of fluorescence was performed in the retinal cross sections processed for immunohistofluorescence. All images were acquired using a fluorescence microscope (20×; ProgRes™ c10; Jenoptic) with a motorized stage (ProScan™ H128; Prior Scientific Instruments Ltd.) connected to an image analysis system (Image-Pro Plus 5.1 for Windows®; Media Cybernetics) with an automatic frame-grabber device (Scope-Pro 5.0 for Windows®; Media Cybernetics). Only one photomontage of one whole cross section spanning the optic nerve was automatically analyzed per animal and time point using a specific subroutine developed for this purpose. In brief, we used the Image-Pro Plus 5.1 for Windows® macro language to apply a sequence of actions and filters to obtain the fluorescent area, maximum and minimum intensity values, average, and standard deviation.

In the first step, the image was converted to 16-bit grayscale, where 0 represents black color (no fluorescence) and 65.535 (2^16) represents white (maximum fluorescence). Then, the contour of the section was drawn on the whole-mount image, and a new image containing the selection and a clear background was created. In the second step, a line was drawn manually in the border between the inner nuclear layer (INL) and the outer plexiform layer (OPL) to separate the external retina (up to the OPL) and internal retina (down to the INL). The contrast of the external retina was inverted, maintaining the contrast of the inner retina untouched to enable a separate layer study. In a third step, 20 equidistant points were automatically dotted along the retinal section (reference point 0 in the optic nerve, 10 dorsal, and 10 ventral). These dots occupied the center of 20 round areas of interest, in which fluorescence density was measured automatically, both in the outer and inner retina. Finally, measurements were exported to a Microsoft Office Excel 2003, (Microsoft Corporation, Redmond, WA) worksheet.

### Statistical analyses

Numeric data were fed into Microsoft® Office Excel 2003 worksheets and exported and analyzed using SigmaStat® 3.1 for Windows® (Systat Software, Inc., Richmond, CA). Descriptive statistics were calculated for each group and different tests were used for comparison between groups. Differences were considered significant when p≤0.05.

### Electroretinographic analysis

To measure the ERG waves and the implicit times, the International Society for Clinical Electrophysiology of Vision criteria were used. The scotopic threshold response (STR) was analyzed for each stimulus; the positive STR (pSTR) was measured from baseline to the “hill” of the positive deflection, approximately 110 ms from the flash onset, and the negative STR (nSTR) was measured from baseline to first “valley” after the pSTR, approximately 220 ms from the flash onset. The paired *t* test was used to compare the ERG responses in the same group of animals before and after light exposure. The Mann–Whitney and Kruskal–Wallis tests were used to compare the ERG parameters between groups of animals.

### Analysis of the retinal ganglion cell population

The Mann–Whitney, Kruskal–Wallis, and Student *t* test were used to compare the numbers of RGCs between groups of animals.

### Fluorescence intensity analysis for protein kinase C alpha and synaptophysin

The Kruskal–Wallis test was used to compare fluorescence intensity between groups of animals.

## Results

### Photoreceptor loss after light exposure

#### Hematoxylin and eosin–stained sections

**Figure 1:**
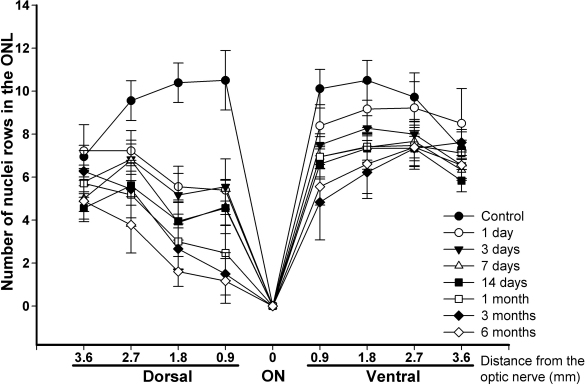
Temporal loss of photoreceptors after light exposure in hematoxylin and eosin–stained retinal cross sections. Graph showing the number of nuclei rows (±standard deviation [SD]) present in the outer nuclear layer (ONL) of six control and six experimental retinas (three right and three left retinas from three animals) at different times after light exposure (ALE) in eight equidistant retinal locations (four dorsal, four ventral). The animals analyzed were chosen randomly from the control and the experimental animals that had been processed to obtain hematoxylin and eosin–stained cross sections. The distance from the optic disc to the retinal location analyzed is shown in the x-axis. Photoreceptor loss was more severe in the dorsal than in the ventral retina. Photoreceptor loss in the dorsal retina was significant at 1 day ALE at all retinal locations (Mann–Whitney test, p<0.001), except at 3.6 mm, where this loss was significant only from 3 days ALE (p<0.001). Photoreceptor loss in the ventral retina was significant at 1 day ALE only at the 0.9 and 1.8 mm locations (p<0.001 and p=0.002, respectively), and at the 2.7 and 3.6 distances, loss was significant from days 3 and 7 ALE, respectively (p<0.001). Photoreceptor loss did not seem to progress from 3 months on (there were no significant differences between the numbers of photoreceptors found 3 or 6 months ALE at any retinal location).

**Figure 2:**
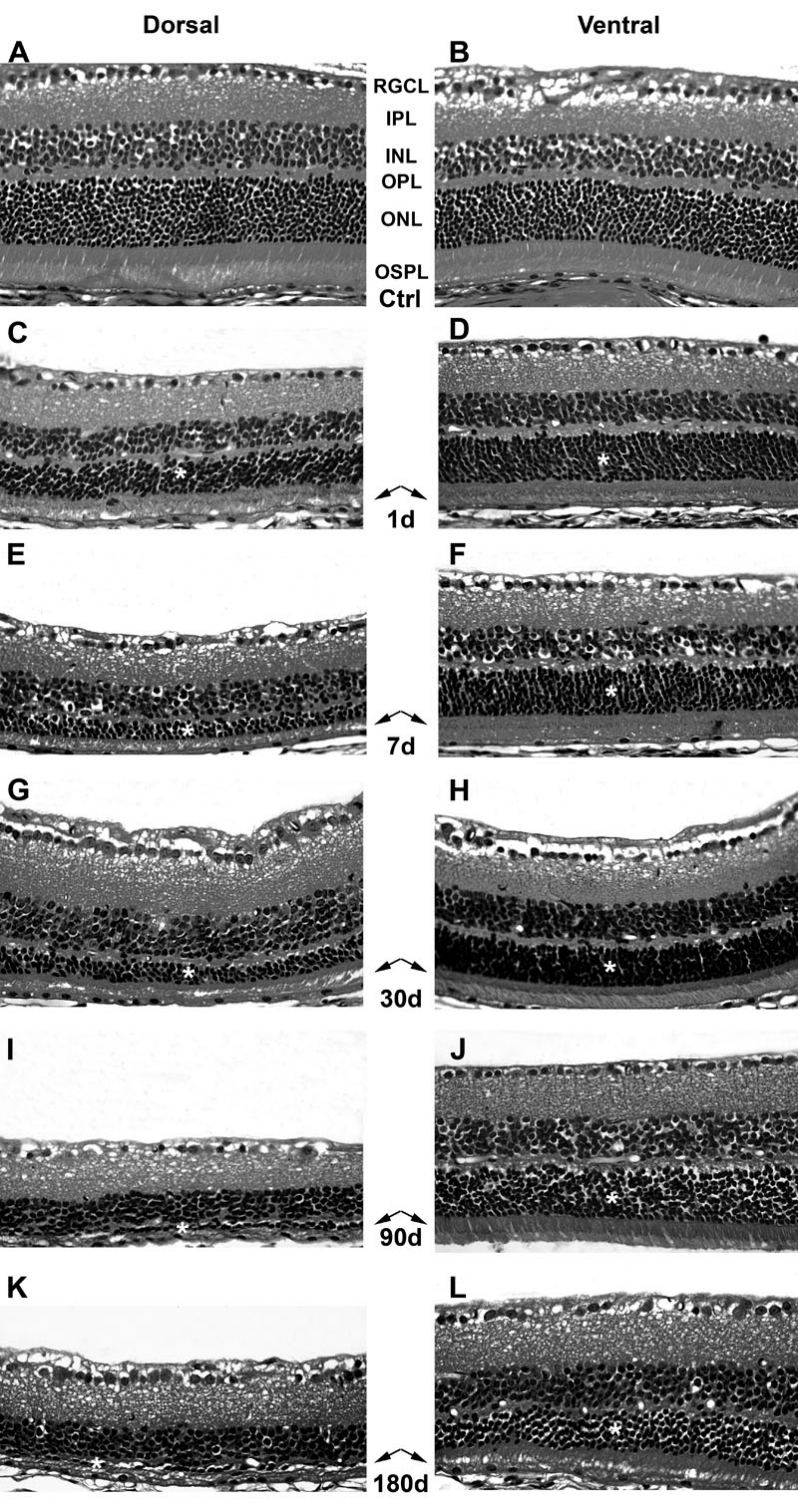
Time course of retinal degeneration after light exposure (ALE). Haematoxylin/eosin stained retinal cross sections from the dorsal (left column) and the ventral (right column) central retina. **A**, **B**: control animals; **C**-**L**: experimental retinas from animals processed at increasing times ALE. **C**, **D**: 1 day ALE; **E**, **F**: 7 days ALE; **G**, **H**: 30 days ALE; **I**, **J**: 90 days ALE and **K**, **L**: 180 days ALE. Abbreviations: RGCL: retinal ganglion cell layer, IPL: inner plexiform layer, INL: inner nuclear layer, OPL: outer plexiform layer, ONL: outer nuclear layer, OSPL: outer segment of photoreceptors layer. Asterisks mark the outer nuclear layer and the arrows point to the outer segments of photoreceptors. Bar=100 μm.

In control animals, the ONL was 9–12 nuclei thick in the peripapillary central retina and 6–8 nuclei thick in the peripheral retina. The INL was 4–5 nuclei thick everywhere in the retina (Figure 1 and Figure 2A,B).

**Figure 3:**
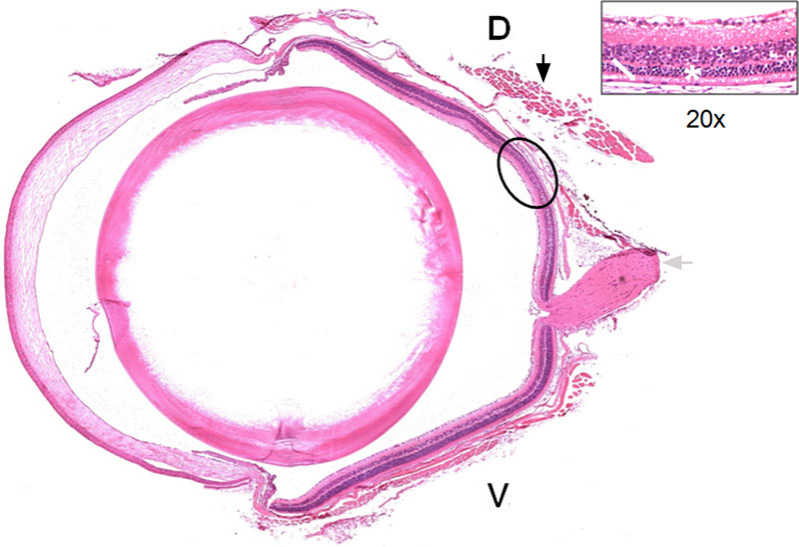
Light damage is more severe in the dorsal retina. Hematoxylin and eosin–stained sagittal cross section of a left eye processed 14 days ALE. The superior rectus muscle (black arrow) and the optic nerve (gray arrow) are observed. The ellipse shows the region most affected by phototoxicity, situated in the dorsal retina at approximately 400 microns from the optic disc. The insert shows a higher magnification of this region, in which the thickness of the outer segments of the photoreceptor layer (white arrow) and the outer nuclear layer (ONL; asterisk) are very much reduced. D: dorsal, V: ventral.

After light exposure, there was a decrease in the thickness of the outer segment photoreceptor layer (OSPL) and the ONL (Figure 1 and Figure 2C-L) at all the survival intervals, with more severe thinning observed for the longer survival intervals. Although the thickness of the OPL decreased proportionally with the thickness of the ONL, the thickness of the INL did not decrease throughout the period of study. The dorsal retina was more severely affected than the ventral retina at all survival intervals and the most peripheral retina did not seem to be affected (Figure 1, Figure 2, and Figure 3). The region of the dorsal retina most severely affected was situated approximately 400 microns from the optic disc (Figure 3). In many of those eyes processed during the first month ALE, we observed an accumulation of subretinal photoreceptor debris in this dorsal region at the time of dissection. There were no differences between the left dilated eyes and the right nondilated eyes at any survival interval, which was also confirmed by the ERG analysis (see below). Therefore, we concluded that pupil dilation did not have an influence on the adverse effects of light on the BALB/c mouse retina.

#### Photoreceptor death is apoptotic

**Figure 4:**
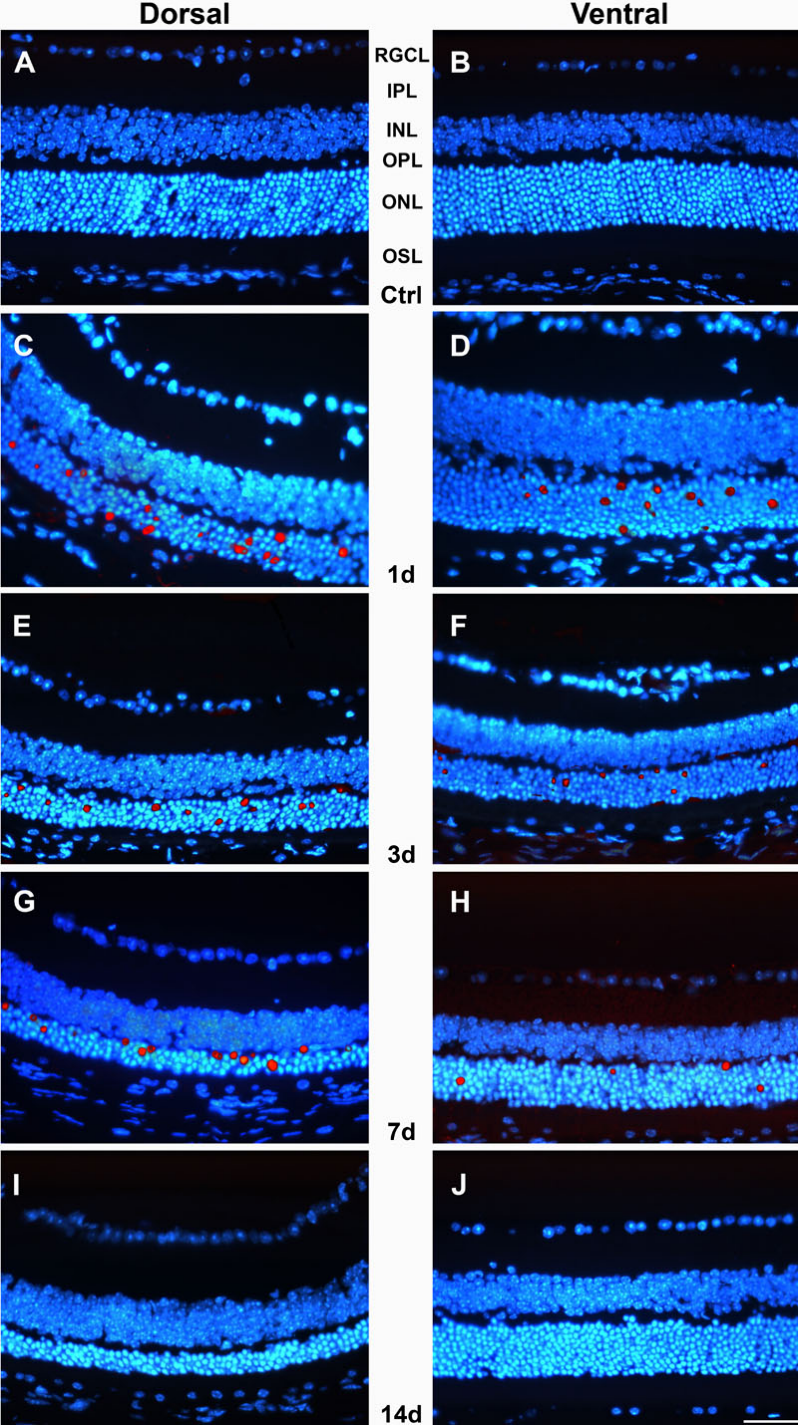
Phototoxicity induces apoptotic photoreceptor death. Merged images (Adobe® Photoshop® CS3, “dissolve” option) showing the TdT-mediated dUTP nick-end labeling (TUNEL)-positive (red) and 4',6-diamidino-2-phenylindole (DAPI)-counterstained (blue) nuclei in retinal cross sections from the dorsal (left column) and ventral (right column) central retinas. **A**, **B**: control animals; **C**-**J**: experimental animals processed at increasing times ALE. **C**, **D**: 1 day; **E**, **F**: 3 days; **G**, **H**: 7 days and **I**, **J**: 14 days. TUNEL positive nuclei are observed in the ONL (mainly in the dorsal retina) of the experimental animals processed 1, 3 and 7 days ALE, but not thereafter. Abbreviations: RGCL: retinal ganglion cell layer, IPL: inner plexiform layer, INL: inner nuclear layer, OPL: outer plexiform layer, ONL: outer nuclear layer, OSPL: outer segment of photoreceptors layer. Bar=100 μm.

TUNEL-positive nuclei (Figure 4) were observed only in the ONL of the central but not the peripheral retina. Analyzed qualitatively, the number of apoptotic nuclei were similar in the right and left eyes, but varied depending on the region of the retina analyzed and the survival period. There were more TUNEL-positive nuclei at early times ALE (1 and 3 days), and these were found mainly in the dorsal retina in the area of maximal cell death (Figure 4C,E,G). At 7 days ALE, there still were some apoptotic nuclei (Figure 4G-H). However, and in spite of the progressing loss of photoreceptors documented in the hematoxylin and eosin–stained sections, no positive TUNEL nuclei were found at longer periods ALE (Figure 4I-J).

### Phototoxicity-induced vascular leakage

**Figure 5:**
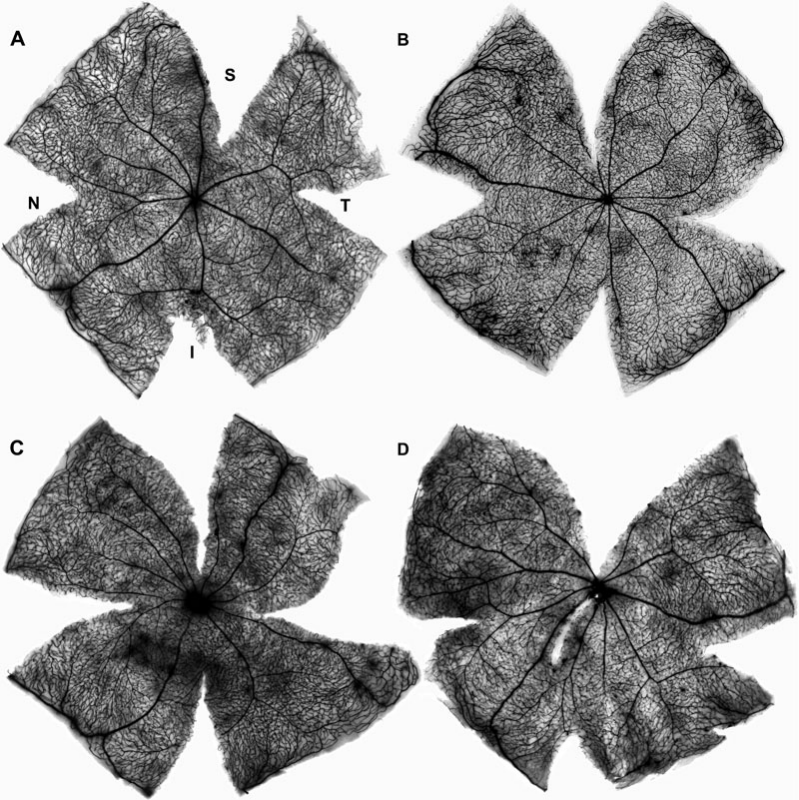
In mice, phototoxicity does not induce vascular leakage. Retinal whole mounts of the left retinas showing the retinal vessels. These animals had received an intravenous horseradish peroxidase (HRP) injection before processing and their retinas were processed for HRP demonstration (Hanker-Yates method). **A**: control mouse, **B**-**D**: photoexposed mice processed at increasing times ALE. **B**: 1 day, **C**: 7 days, **D**: 30 days ALE. Although in some cases a few areas of discrete HRP leakage around the vessels due to retinal damage during dissection are observed, a diffuse HRP staining, indicative of vascular leakage, was never detected. Abbreviations: S: superior, I: inferior, N: nasal, T: temporal.

We have documented recently that in the rat, light exposure induces vascular leakage in an arciform area located in the dorsal retina. Vascular leakage occurs during the first week and first month ALE in albino and pigmented rats, respectively [5,6]. Importantly, this arciform area of vascular leakage is where the highest sensitivity to light was observed. To document whether phototoxicity also caused vascular leakage in mice, HRP was intravenously injected in control (Figure 5A) and photoexposed mice (Figure 5B-D) and the retinas processed for HRP. These data demonstrated that in mice, there does not appear to be vascular leakage after light exposure, at least with the experimental conditions and times ALE studied here.

### Retinal function: electroretinographic responses

**Figure 6:**
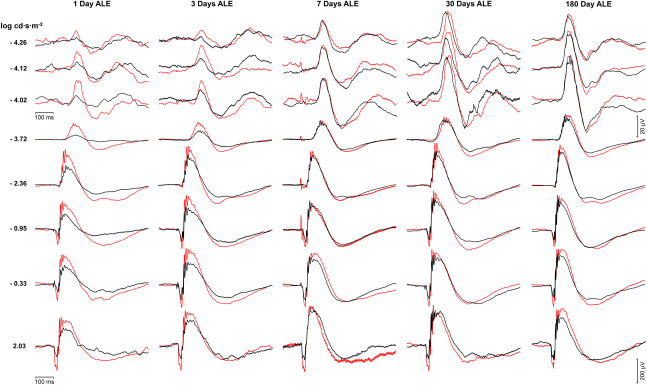
Changes in the electroretinogram responses after light exposure. Electroretinographic (ERG) responses recorded from the left eyes of control animals before light exposure (red lines) and experimental animals 1, 3, 7, 30, or 180 days after light exposure (ALE; black lines). The three first rows correspond with the scotopic threshold response (STR) waves, while the rest correspond with the a- and b-waves. For convenience, the responses recorded in control animals before light exposure have been superimposed over the responses recorded in experimental animals. For details on control animals, see text.

**Figure 7:**
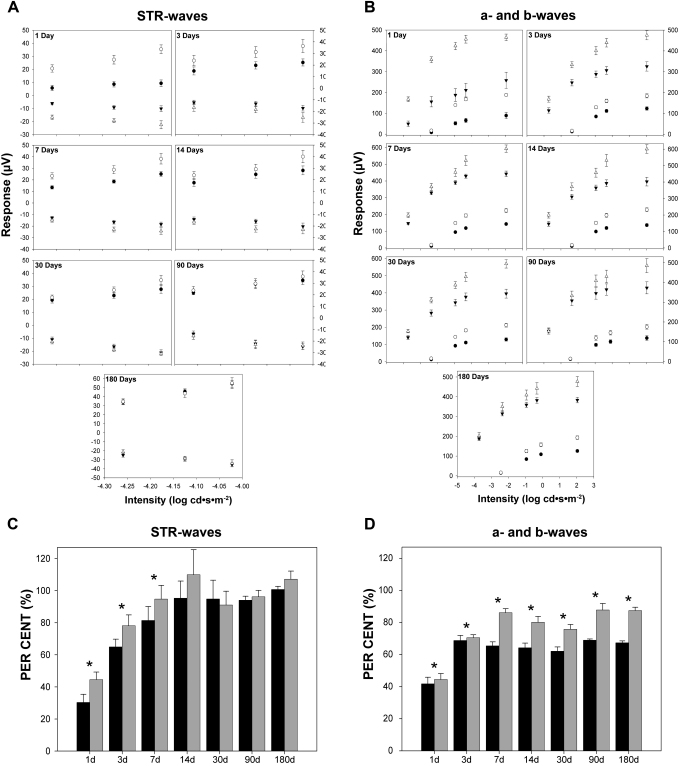
Quantification of the electroretinographic responses after light exposure. **A**: Mean amplitudes of the positive scotopic threshold response (pSTR; circles) and negative STR (nSTR; triangles; ±standard error of the mean [SEM]) obtained using different light stimuli from control (open circles and triangles) and experimental animals (closed circles and triangles) at the different periods studied: 1 (n=8), 3 (n=8), 7 (n=16), 14 (n=12), 30 (n=13), 90 (n=10), and 180 days (n=10) after light exposure (ALE). The STR waves are affected by light exposure, but recover almost completely by 14 days ALE. **B**: Mean amplitudes of the a- (circles) and b- (triangles) waves (±SEM) obtained using different light stimuli from control (open circles and triangles) and experimental animals (closed circles and triangles) at the different periods studied (same as in **A**). The a- and b- waves are affected by light exposure, and while the a-wave is reduced at all times points, at 90 and 180 days ALE the b- wave is similar to control values, except for the highest intensity stimulus (2.03 log). **C**, **D**: Percentage of electroretinographic (ERG) waves considered 100% of their control values at the different analyzed time points **C**: pSTR (black bars) and nSTR (gray bars) waves. **D**: a- (black bars) and b- (gray bars) waves. Asterisks: statistically significant compared to control values (Kruskal–Wallis test, p≤0.05)

The baseline ERG responses obtained before light exposure were normal and similar in both eyes in all the animals studied. The same animals were recorded prior and ALE in the groups of animals processed for up to 30 days ALE, while age-matched animals were used as control animals in the groups processed 90 and 180 days ALE to account for any possible aging effects. The scotopic ERG responses of the photoexposed animals varied depending on the survival interval, the wave, and the stimuli, but were similar in both right and left (dilated) eyes (Figure 6 and Figure 7). On the other hand, the photopic responses were diminished but varied greatly between animals and thus were not conclusive (data not shown).

The STR is the ERG wave that reflects the functionality of the inner retina (Figure 6, three first rows; Figure 7A,C). One day ALE, the pSTR and nSTR were reduced to approximately 35% of the values obtained before light exposure (baseline), and this reduction was statistically significant (paired *t* test, p<0.05; Figure 7C). Three days ALE, the pSTR and nSTR had recovered compared to 1 day ALE, but only to approximately 65% and 78% of their baseline values, respectively (Figure 7A,C). The absolute values of pSTR were significantly smaller than the baseline records (paired *t* test, p<0.05; Figure 6C), while the values of the nSTR were significantly smaller for only one of the three stimuli studied (the −4.02 log light unit stimulus; Figure 7A). Seven days ALE, the recovery of the pSTR and nSTR continued, reaching approximately 80% and 90% of the baseline values, respectively (Figure 7C). This reduction was still significant (paired *t* test, p<0.05) for the pSTR for all the stimuli studied, but for the nSTR this was only significant for the −4.12 log light unit stimulus (Figure 7A). From 14 until 180 days ALE, the STR waves were not significantly different from control recordings (baseline or age-matched controls; Student’s *t* test, p>0.05) or from animals processed at earlier times ALE (Kruskal–Wallis, p>0.05; Figure 7A,C).

The a- and b- waves of the ERG reflect the functionality of the photoreceptors and the bipolar and/or Müller cells, respectively (Figure 6, five last rows; Figure 7B,D). One day ALE, there was a reduction of the a- and b-waves (paired *t* test, p<0.05) to approximately 40% of baseline values (Figure 7D). Three days ALE, both waves recovered compared with the previous time point, but still were significantly lower than the baseline values (70%, paired *t* test, p<0.05; Figure 7D). At seven days ALE, their amplitudes continued to be significantly reduced (paired *t* test, p<0.05), although they had recovered to 65% (a-wave) and 90%, (b-wave) of their baseline amplitudes (Figure 7B,D). Fourteen days ALE, the a- and b-waves were reduced to approximately 65% and 80% of their baseline values. This reduction was significant compared to baseline (paired *t* test, p<0.05), but not compared to 3 or 7 days ALE in the case of the a-wave or to 7 days ALE in the case of the b-wave (Kruskal–Wallis, p>0.05; Figure 7D). Thirty days ALE, the amplitudes of the a- and b-waves were still reduced to approximately to 65% and 80%, and these reductions were statistically significant compared to controls (paired *t* test, p<0.05), although not significantly different from those observed 14 days ALE (Kruskal–Wallis, p>0.05; Figure 7D). Ninety and 180 days ALE, the a-wave was still maintained at 70% of control values (paired *t* test, p<0.05 at 90 days, p<0.001 at 180 days), while the b-wave had recovered to 90% of controls. This value was not different from that observed from 7 to 30 days ALE (Figure 7D). At these time points, the reduction of the b-wave was significantly smaller only for the most intense flash stimulus (Student’s *t* test, p≤0.05; Figure 7B).

In conclusion, our data indicate that phototoxicity causes a transient functional impairment of the inner retina, because after a significant decrease during the first 7 days ALE, the STR recovers, regaining its baseline values at 14 days. Photoreceptor functionality is permanently affected, as the a-wave impairment lasts until 180 days ALE, although it showed some recovery during the period of the study. Bipolar and/or Müller cells do not regain full functionality because, even though the b-wave improves almost to control values, at 90 and 180 days ALE the response to the brightest stimulus is still affected (Figure 6 and Figure 7).

### Compensatory changes: protein kinase C α and synaptophysin expression levels after light exposure

Because our functional data indicated a partial recovery of the a-wave, an almost complete recovery of the b-wave in the first 3 months ALE, and a complete recovery of the STR waves in the first 14 days, we speculated that there could be compensatory retinal changes to account for the functional recovery. To assess this possibility, we studied the expression pattern and levels of two proteins: PKCα, a protein kinase associated with bipolar cells, and synaptophysin, an integral membrane protein expressed in the presynaptic vesicles [34].

**Figure 8:**
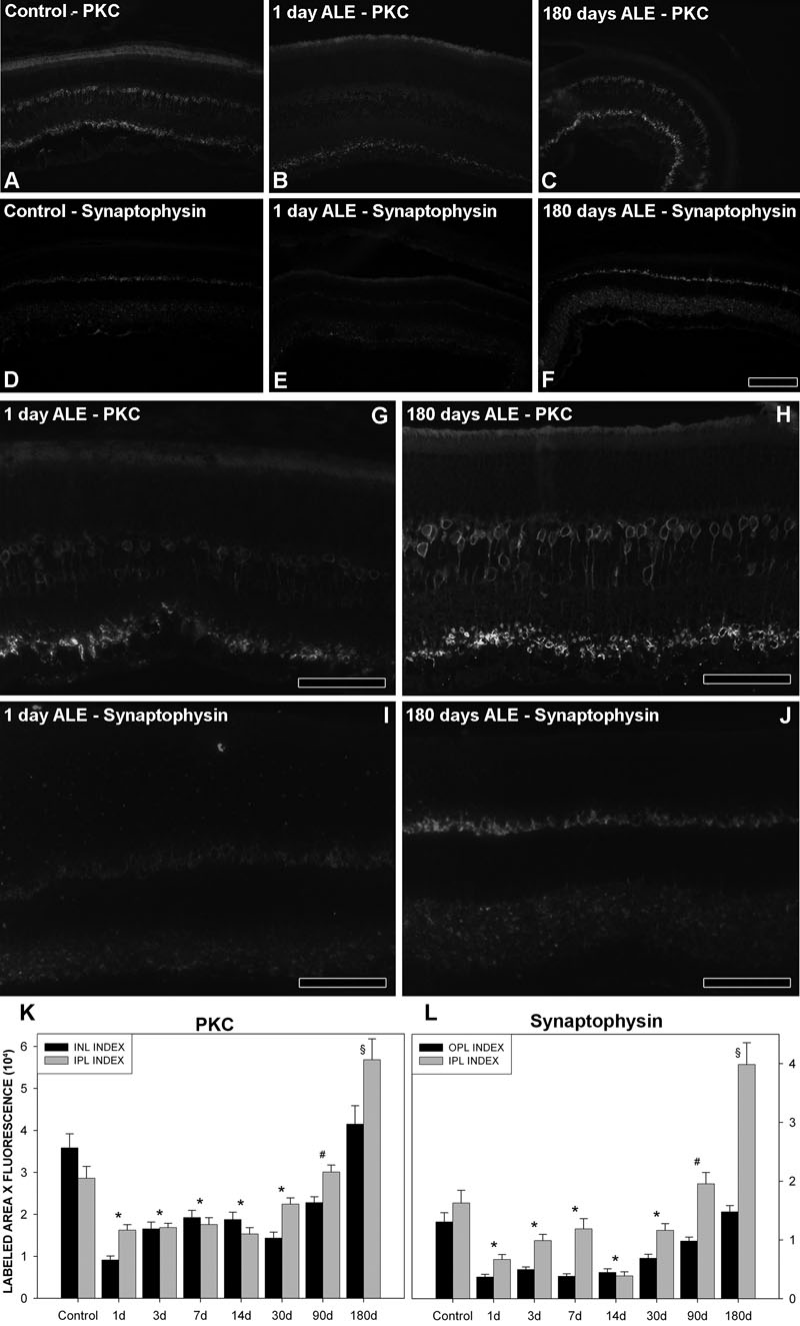
Changes in the expression level of protein kinase C alpha (PKCα) and synaptophysin after light exposure (ALE). Retinal cross sections reacted with antibodies to anti-PKCα and anti-synaptophysin. **A**-**D**: control animals, **B**, **E**, **G**, **I**: animals processed at 1 day ALE, **C**, **F**, **H**, **J**: animals processed 180 days ALE. **B**, **C**: PKCα signal, magnified in **G**, **H**. **E**, **F**: synaptophysin signal, magnified in **I**, **J**. Compared to control retinas, there is a decrease in the expression of these two proteins 1 day ALE and an increase 180 days ALE. **K**-**L**: Graphs showing the relative levels of fluorescent signal per labeled area of PKCα (**K**) and synaptophysin (**L**) in control and experimental retinas ALE. Asterisks: both indexes were statistically significant compared to control. # INL (**K**) and OPL (**L**) index was statistically significant compared to control, § IPL (**K** and **L**) index was statistically significant compared to control (Kruskal–Wallis test p≤0.05). Signal intensity level is expressed in arbitrary units, where 0 would be no fluorescence (black) and 65,335 (6×10^4^) would be the maximum fluorescence (white). See methods for further explanations. Abbreviations: IPL: inner plexiform layer. INL: inner nuclear layer. OPL: outer plexiform layer. Bar=100 µm.

In control retinas, PKCα is expressed in the inner plexiform layer (IPL) and the INL (Figure 8A). Quantification of the intensity of fluorescence per area (Figure 8K) showed that this expression is around 3.6±0.3 (×10^4^) and 2.9±0.3 (±10^4^) arbitrary units for the INL and IPL, respectively. Synaptophysin is expressed in the OPL and IPL (Figure 8D), with a fluorescence intensity of 1.3±0.2 (×10^4^) and 1.6±0.2 (×10^4^) arbitrary units, respectively (Figure 8L).

One day ALE, the PKCα signal decreases in both layers below control values (INL: 0.9±0.09 [×10^4^]; IPL: 1.6±0.13 [×10^4^] arbitrary units; Figure 8B,G,K). From this time forward, PKCα expression recovers gradually, and by 90 days ALE in the IPL and 180 days ALE in the INL, reaches values similar to those found in control animals (Kruskal–Wallis, p>0.05). In the IPL, the PKCα signal at 180 days is significantly higher than in control retinas (5.7±0.49 [×10^4^] arbitrary units, Kruskal–Wallis, p<0.05; Figure 8C,H,K).

The behavior of synaptophysin follows a similar trend. One day ALE, its signal diminishes below control values in the IPL (0.36±0.04 [×10^4^]) and OPL (0.66± 0.08 [×10^4^]) (Kruskal–Wallis, p<0.05; Figure 8E,I,L). In the OPL its signal is kept at the same lower levels up to day 14 ALE, and thereafter its expression augments until reaching normal values at day 180 ALE (Kruskal–Wallis, p>0.05; Figure 8L). In the IPL, however, after reaching its lowest level at day 14 ALE, the synaptophysin signal continuously increases and by day 180 ALE, its value is significantly higher than in control animals (3.9±0.3 [×10^4^] arbitrary units, Kruskal–Wallis, p<0.05; Figure 8F,J,L).

### Retinal ganglion cell population and spatial distribution

**Figure 9:**
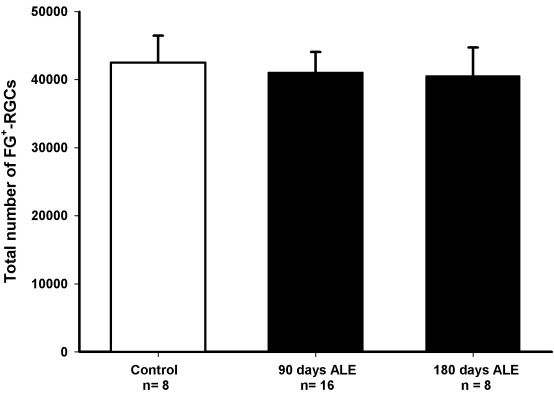
Number of Fluoro-Gold-traced retinal ganglion cells in control and experimental retinas at 90 and 180 days after light exposure. The mean numbers (±standard deviation [SD]) of retinal ganglion cells (RGCs) in these groups were 42,477±3,953, 41,015±3,045, and 40,566±4,146, respectively. The numbers of RGCs were not significantly different between control (white bars) and experimental retinas (black bars) at any time point analyzed (Mann–Whitney, p>0.05), or between the two groups of experimental animals (*t* test, p>0.05) or the left and right retinas counted in each group analyzed (*t* test for control and 90 days after light exposure [ALE] groups and Mann–Whitney test for the 180 days ALE group, p>0.05). n=number of retinas.

**Figure 10:**
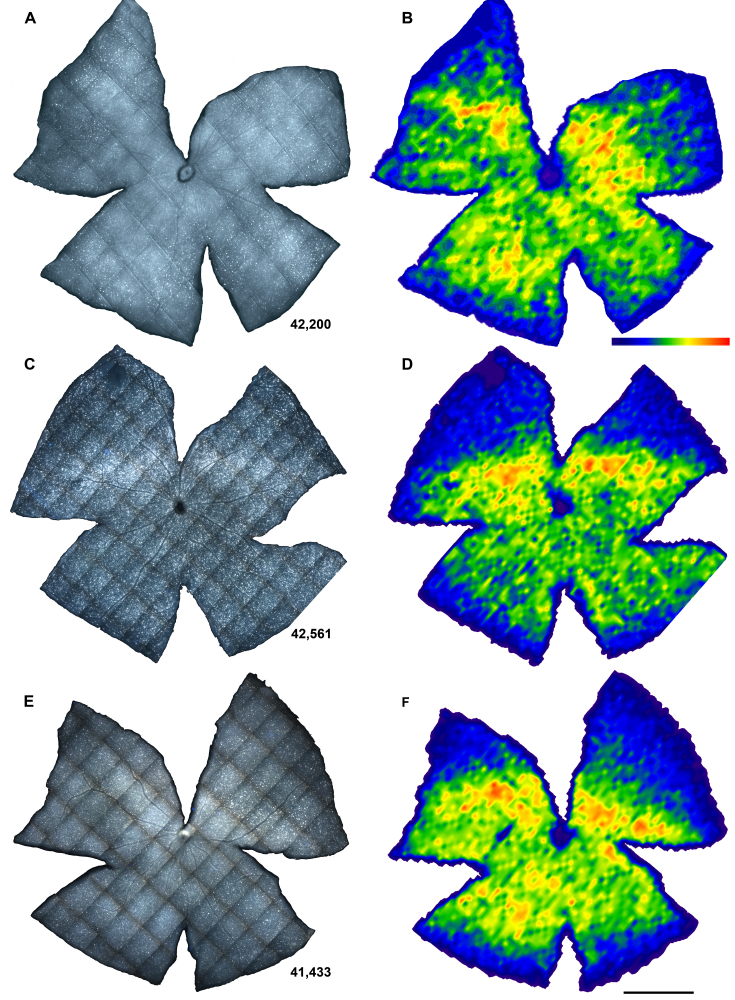
Distribution of retinal ganglion cells (RGCs) in control and experimental retinas. **A**, **C**, **E**: Retinal photomontages showing fluoro-gold traced RGCs and **B**, **D**, **F**: their corresponding isodensity maps. **A**, **B**: control mouse; **C**, **D**: experimental retina processed 90 days ALE; **E**, **F**: experimental retina processed 180 days ALE. Isodensity maps are filled contour plots generated by assigning to each one of the subdivisions of each individual frame a color code according to its RGC density value within a color-scale range that goes from 0 (purple) to 5,700 or higher (red) RGCs/mm^2^ (B bottom). The numbers of RGCs in these representative retinas are shown at the bottom of **A**, **C**, **E**. Bar=1 mm.

In albino rats, phototoxicity induces a delayed death of RGCs, which affects the whole retinal surface, although there are specific sectors devoid of RGCs. To analyze whether there is also RGC death in albino mice, we automatically quantified FG-labeled RGCs and assessed their spatial distribution. The mean number±standard deviation (SD) of FG-traced RGCs in control animals was 42,477±3,953 cells (Figure 9 and Figure 10A). RGCs were distributed in a nonhomogeneous way. An area of maximal RGC density was observed above the optic nerve horizontally from the nasal to the temporal retina (Figure 10B), as described in normal mice [33,35].

The population of RGCs decreased slightly, with lower numbers present at 180 than at 90 days ALE. However, this diminution was not significant (Mann–Whitney, p>0.05; Figure 9 and Figure 10C,E). In addition, the distribution of the FG-traced RGCs in light-exposed mice was similar to the distribution observed in control retinas (Figure 10D,F). In conclusion, although the number of FG-traced RGCs decreased with time, there was not a significant RGC loss during the first 180 days ALE.

## Discussion

To our knowledge, this is the first study that analyzes in detail the temporal course (from 1 to 180 days) of the functional, anatomic, and compensatory changes taking place in the mouse retina following a phototoxic insult. Anatomically, photoreceptors die mostly in the dorsocentral retina, sparing the periphery. Interestingly, the inner retina is not affected. These data correlate with the ERG analysis, which showed that the a-wave is permanently affected, while the b-wave (which reflects bipolar cell function) recovers 90% of its amplitude and the STR waves (which correspond to RGC function) recover their basal values. All these data, together with the changes observed in PKCα and synaptophysin expression, show that light exposure in mice results in retinal remodeling changes that allow the functional recovery of the inner retina.

We exposed mice to 24 h of cold white light (fluorescent tubes situated in the ceiling above the cages) to broadly reproduce the lighting conditions used in our previous experiments in rats [5,6]. Because retinal phototoxicity depends on light spectra and the shorter wavelengths usually cause more severe damage [3,36,37], other authors have preferentially used blue or green light to induce retinal damage [38,39]. However, we used cold white fluorescent light because this does not have a heating effect, contains all wavelength spectra, and better reproduces the normal lighting conditions of the animals in nature or captivity.

In this study, white light damage induced photoreceptor degeneration by apoptosis and an initial loss of retinal function. Photoreceptor death was quicker and more severe in the dorsal than in the ventral retina, progressed during the time of study (6 months), and in this species, did not reach the retinal periphery. However, the functional impairment recovered partly with time and the degree of recovery was found to depend on the neuronal population under study. Thus, while the STR waves recovered completely and the b-wave (bipolar cells) almost reached control values, indicating the functional recovery of the inner retina, the a-wave (linked to photoreceptor function and reflecting the outer retinal function) was permanently impaired.

These data allow us to conclude that with the experimental conditions used, there are differences in the pattern of light-induced retinal degeneration between albino rats and mice. It should be noted, however, that the duration of light exposure differed between the two models studied. The temporal course of the retinal degeneration in albino rats exposed to the same light intensity (3,000 lx) but during 48 h instead of 24 h, included the following events: i) complete abolishment of the ERG waves from the first day ALE; ii) rupture of the blood-retinal barrier and vascular leakage in an area of the dorsal retina, the “arciform” area; iii) apoptotic death of photoreceptors, commencing in the dorsal retina and later spreading to the whole retinal surface; iv) changes in the nerve fiber layer, marking the degeneration of RGCs; v) RGC axons being dragged by displaced retinal vessels; and vi) significant RGC loss by 6 months ALE [5]. In this study in albino mice, we also observed apoptotic death of photoreceptors starting in the dorsal retina. In this study, however, photoreceptor loss was slower and less severe than in the rat and did not affect the whole retina [5]. While it could be that the difference is due to the shorter length of light exposure, we do not think that this is the primary reason because in previous experiments with albino mice, 48 h of exposure caused devastating effects (data not shown) that were much more severe than those observed in the rat. Thus, given that 48 h of light exposure causes disproportionate damage in albino mice than in albino rats, our data would suggest that there are other differences in the phototoxic response between these species. In addition, while in mice, pupil dilation does not seem to affect the degree of the damage, in rats, mydriasis quickens photoreceptor death during the first month ALE [5]. Collectively, these differences very likely reflect strain differences in light sensitivity [25,29].

Differences between rat and mouse responses to light exposure have been previously shown, and are proposed to have a genetic origin. For example, a sequence variant in the *RPE65* gene has been shown to determine the susceptibility to light damage in different mouse strains [40-42], as well as in mice with inherited retinal degeneration [43]. In rats, however, light damage did not seem to correlate with RPE65 protein levels [44], and thus it has been proposed that there are other genes involved in light toxicity that may differentially influence the effect of the *RPE65* gene across species [45].

Another difference between albino rats and mice is that in the latter, we did not observe vascular leakage. The retinal degeneration triggered by light in rats ends with the death of RGCs [5,6], which is significant by 6 months ALE [5], and RGC loss is clearly quantifiable when photoreceptor degeneration is complete. In mice, phototoxicity damage does not appear to reach the RGCs, at least up to 6 months ALE. This agrees with the ERG data, which show that the STR waves recover completely by 14–30 days ALE (see below). Nevertheless, because photoreceptor loss ALE in mice develops at a slower pace than in rats, it is possible that in mice, RGC degeneration also occurs later.

The ERG a- and b- waves reflect photoreceptor functionality and thus are severely affected after light-induced retinal damage [17,46,47]. Depending on the severity of the damage, the a- and b-waves may recover partially or completely afterwards [48], with a decrease in the a-wave/b-wave ratio that is also indicative of the features of retinal light damage [17]. Comparable reductions of the a- and b-waves have been shown to be the result of retinal pigment epithelial (RPE) damage, while a smaller reduction of the b-wave is the result of photoreceptor damage [49]. We have observed a large decrease of both waves during the first days ALE that coincides with the massive death of photoreceptors detected at days 1 and 3 ALE. At later times ALE, the ratio between both waves changes, the reduction of the b-wave being smaller than that of the a-wave. Thus, it is likely that in our model there is an impairment of the RPE during the first three days ALE, which is followed by photoreceptor impairment at later time points. Because the RPE cells will phagocyte the dead photoreceptors [3,50,51], their impairment ALE may be responsible for the subretinal photoreceptor debris accumulation that is observed at early times ALE. Subretinal deposits have been observed previously in animal models of light toxicity [3,52], inherited retinal degeneration [53], and human age related macular degeneration (ARMD) [54], and thus might be common to all the diseases that present with rapid photoreceptor death.

We observed a decrease of photoreceptor functionality down to 60%–65% of the control values. Because the dorsocentral retina is the only part of the retina that shows photoreceptor loss, the surviving photoreceptors could be responsible for the maintenance of 60%–65% of the a-wave after damage. As there has been some photoreceptor loss, however, there is a permanent decrease of the a-wave amplitude. Sugawara et al. [17] studied the a-, b-, and STR waves 7 days ALE in the albino rat, and were able to show that there is a correlation between b- and STR wave characteristics and the amount of photoreceptor loss. We have observed an early impairment of the b- and STR waves that may correlate with photoreceptor loss. Later, there is a complete recovery of these waves that may occur because photoreceptor loss in this animal model is not as severe, and compensatory mechanisms can come into play. In accordance with this, in albino rats, which suffer a much more severe photoreceptor loss ALE, there is a complete and permanent abolition of the ERG response [5].

In photoexposed albino mice, photoreceptor degeneration peaks at days 1–3, decreasing by day 7, when some TUNEL-positive nuclei are still found; by day 15, no more apoptotic nuclei are detected. If the outer retina is affected, the signal that is sent to the bipolar cells (the next layer of neurons) would be also affected, and consequently, the response of these second-order neurons would be impaired. Two articles have studied the functional response of inner retina ALE in rat [17] and mice [48] and have reported that the functionality of the inner retina was less affected than the outer one. This was demonstrated by analyzing the STR at 7 days ALE [17] or by studying the oscillatory potentials at different times ALE [48]. Richards et al. [48] observed functional compensatory changes in mice in the inner layers 90 days ALE and in specific bipolar functionality at 180 days after the insult. It is worth highlighting that this is the first study wherein the STR responses of the albino mouse retina are analyzed ALE. Here, we have demonstrated that the STR is affected immediately ALE, but starts to recover slowly from 3 days and is close to baseline values by 14 days ALE. The temporal differences in the recovery of the inner layers’ functionality observed between our data and those from Richards et al. [48] might be explained by the different methods used. Richards et al. [48] studied the oscillatory potentials, which are probably generated by amacrine cells [55], while we analyzed the STR, which is associated mainly with RGCs [10,12,14,15]. Another explanation could be the differences between the phototoxicity models, because Richards et al. [48] exposed mice to an intensity of 150–175 lx for 20 days, while our model was more acute.

The temporal impairment of the b- and STR waves observed in this study suggests that there are retinal compensatory changes ALE [48]. In this study, if light damage had only affected photoreceptors, the STR should have been fully recovered at 3 days ALE, because it is at this time point that the a-wave is stabilized to the value that will be maintained until the end of the experiment. However, the STR was statistically recovered at 14 days ALE, and this may be interpreted as an indication that the circuitry of other layers that ends in the RGCs must undergo compensatory changes that take longer to complete than those at the outer retinal level. One of these compensatory changes could be synaptogenesis or an amplification of the existent synapses, as this has been observed in various studies that show remodeling of the retina ALE or in other types of photoreceptor degeneration [1,34,56-59]. Here, we also show that PKCα and synaptophysin, proteins related to compensatory mechanisms, are downregulated up to 90 days ALE, recovering their normal values at 180 days ALE in the INL and IPL and above control values in the IPL and OPL, respectively. Because PKCα is a protein kinase associated with retinal bipolar cells (with a key regulatory role in a variety of cellular functions), and synaptophysin is an integral membrane protein situated in the presynaptic vesicles that contain neurotransmitters, the recovery of these proteins may suggest an improvement of bipolar cell function. However, the recovery of PKCα and synaptophysin was slow, and thus did not match the fast recovery of the b-wave or the STR waves; therefore, it may constitute only a part of a more complex compensatory retinal mechanism that involves other retinal changes, such as, for example, the expression of glutamate or other inhibitory receptors, as this has been shown during retinal development [60,61] or degeneration [59,62,63]. Future experiments will elucidate this possibility.

In conclusion, our data show that light exposure causes photoreceptor death in albino mice, which is limited and followed by the recovery of inner retinal function. We also show that in this model (in contrast to our previous study in adult rats), there is preservation of the architecture of the inner retina, and that the RGCs do not seem to be affected. Taken together, these findings suggest that when photoreceptor loss is not too severe, the inner retina may only suffer limited damage and can therefore recover its function. Although further electron microscopy and/or behavioral studies may be required to confirm this hypothesis, our present results give hope to retinal therapies aimed to replace only the lost photoreceptors, such as photoreceptor transplantation or retinal prosthesis implantation.
